# Design and Application of a Solar Mobile Pond Aquaculture Water Quality-Regulation Machine Based in Bream Pond Aquaculture

**DOI:** 10.1371/journal.pone.0146637

**Published:** 2016-01-20

**Authors:** Xingguo Liu, Hao Xu, Zhuojun Ma, Yongjun Zhang, Changfeng Tian, Guofeng Cheng, Haisheng Zou, Shimin Lu, Shijing Liu, Rong Tang

**Affiliations:** 1 Key Laboratory of Fishery Equipment and Engineering, Ministry of Agriculture, Shanghai 200092, China; 2 Fishery Machinery and Instrument Institute, Chinese Academy of Fishery Science, Shanghai 200092, China; The Ohio State University, UNITED STATES

## Abstract

Bream pond aquaculture plays a very important role in China’s aquaculture industry and is the main source of aquatic products. To regulate and control pond water quality and sediment, a movable solar pond aquaculture water quality regulation machine (SMWM) was designed and used. This machine is solar-powered and moves on water, and its primary components are a solar power supply device, a sediment lifting device, a mechanism for walking on the water’s surface and a control system. The solar power supply device provides power for the machine, and the water walking mechanism drives the machine’s motion on the water. The sediment lifting device orbits the main section of the machine and affects a large area of the pond. Tests of the machine’s mechanical properties revealed that the minimum illumination necessary for the SMWM to function is 13,000 Lx and that its stable speed on the water is 0.02–0.03 m/s. For an illumination of 13,000–52,500 Lx, the sediment lifting device runs at 0.13–0.35 m/s, and its water delivery capacity is 110–208 m^3^/h. The sediment lifting device is able to fold away, and the angle of the suction chamber can be adjusted, making the machine work well in ponds at different water depths from 0.5 m to 2 m. The optimal distance from the sediment lifting device to the bottom of the pond is 10–15 cm. In addition, adjusting the length of the connecting rod and the direction of the traction rope allows the SMWM to work in a pond water area greater than 80%. The analysis of water quality in Wuchang bream (*Parabramis pekinensis*) and silver carp (*Hypophthalmichthys molitrix*) culture ponds using the SMWM resulted in decreased NH_3_^+^–N and available phosphorus concentrations and increased TP concentrations. The TN content and the amount of available phosphorus in the sediment were reduced. In addition, the fish production showed that the SMWM enhanced the yields of Wuchang bream and silver carp by more than 30% and 24%, respectively. These results indicate that the SMWM may be suitable for Wuchang bream pond aquaculture in China and that it can be used in pond aquaculture for regulating and controlling water quality.

## Introduction

Freshwater pond aquaculture is the major culture method in China; the freshwater aquaculture ponds have an area of 2,623,176 ha, and freshwater aquaculture produced 19,887,462 t in 2014 in China [1]. Blunt-nose bream is one of the most dominant seven fish species in freshwater aquaculture of China, which provide much more fish production than any other country [2]. The bream has another name of Wuchang bream (*Parabramis pekinensis*). However, the intensive pond culture of the fish has resulted in some serious problems including pollution in water column and sediment. As the development of aquaculture, concerns regarding the possible effects of the ever-increasing amount of aquaculture waste have increased. It has been reported that 10%–20% of fish food is not eaten by bream but rather directly deposited at the bottom of the pond. Moreover, the food eaten by fish represents only 20%–25% of the nitrogen and 25% –40% of the phosphorus in the food used for growth, and the remaining 75%–80% of the nitrogen and 60%–75% of the phosphorus are discharged into the pond environment as faeces in Jiangsu and Zhejiang areas of China [3]. The presence of a significant amount of organic matter in pond sediment not only leads to polluted water but also produces excess amounts of ammonium, nitrite, hydrogen sulphide and other harmful substances, which can influence the safety of aquaculture [4]. In contrast, phytoplankton, which play a vital role in maintaining the stability of the pond ecosystem, not only need to assimilate large amounts of nitrogen, phosphorus, sulphur and other nutrients but also act as the primary source of dissolved oxygen in the water and are an excellent natural food [5]. Some other researchers have found that the soluble phosphorus concentration in sediment is 10–50-fold higher than the level observed in water and that the phosphorus in the sediment can be discharged into the water by agitation, which helps promote the growth of microbes and phytoplankton and improves the material conversion efficiency of aquaculture systems [6–8]. As the environmental problems of aquaculture emerged in the 1990s, people began to pay attention to aquaculture pond sediment and water quality control technologies. Some scholars believe that pond sediment management is a breakthrough technology that promotes pond farming [9]. Currently, to improve the environment in aquaculture ponds, methods for processing sediment are often selected from mechanical dredging, bottom aeration, probiotics, and chemical treatments [10]. Although mechanical dredging can be used in almost every aquaculture pond, it requires a large area for discharging mud, and the nutrients in the sediment can hardly be reused [11]. Bottom aeration can effectively improve the amount of oxygen dissolved in the sediment and accelerate the decomposition of organic matter but it is expensive, and aeration pipes can become blocked over time [12]. Probiotics can accelerate the sediment mineralization process and utilize some harmful substances [13], but the process will consume large amount of dissolved oxygen and may therefore lead to fish hypoxia. The chemical method is rapid, but it is easy to leave drug residues behind, which can cause secondary pollution [14]. In addition, aerators, till water machines, and surge wave machines, among others, could also improve the pond sediment to some extent, but their effects are weaker because these devices are arranged in fixed positions [10]. In addition, a great deal of power is required to drive this material, which undoubtedly significantly increases the cost of aquaculture.

To solve the aforementioned problems, we introduce a solar mobile water quality-regulation machine (SMWM) in this manuscript. This machine walks regularly on the water on sunny days, and the flocculent sludge and water from the bottom of the pond are cast to the surface water. The mechanical properties and working effects of this machine were analysed to provide an efficient, energy-saving device for controlling the quality of aquaculture pond water.

## Methods

### 2.1 The SMWM design proposition

The SMWM is a new type of equipment for water quality control that travels on the surface of a pond in a specific direction and is powered by solar energy under the precondition that there is abundant sunlight. With a sediment lifting device, the machine can absorb flocs from the pond bottom and release them into the upper layer of water, where the dissolved oxygen produced by the photosynthesis of phytoplankton is used to promote the decomposition of the flocculent organic matter in the bottom sediment and release nutrient elements, such as nitrogen and phosphorus, from the bottom sediment, thus achieving the goal of controlling the water quality and bottom sediment while improving the primary productivity of the water.

**Fig 1:**
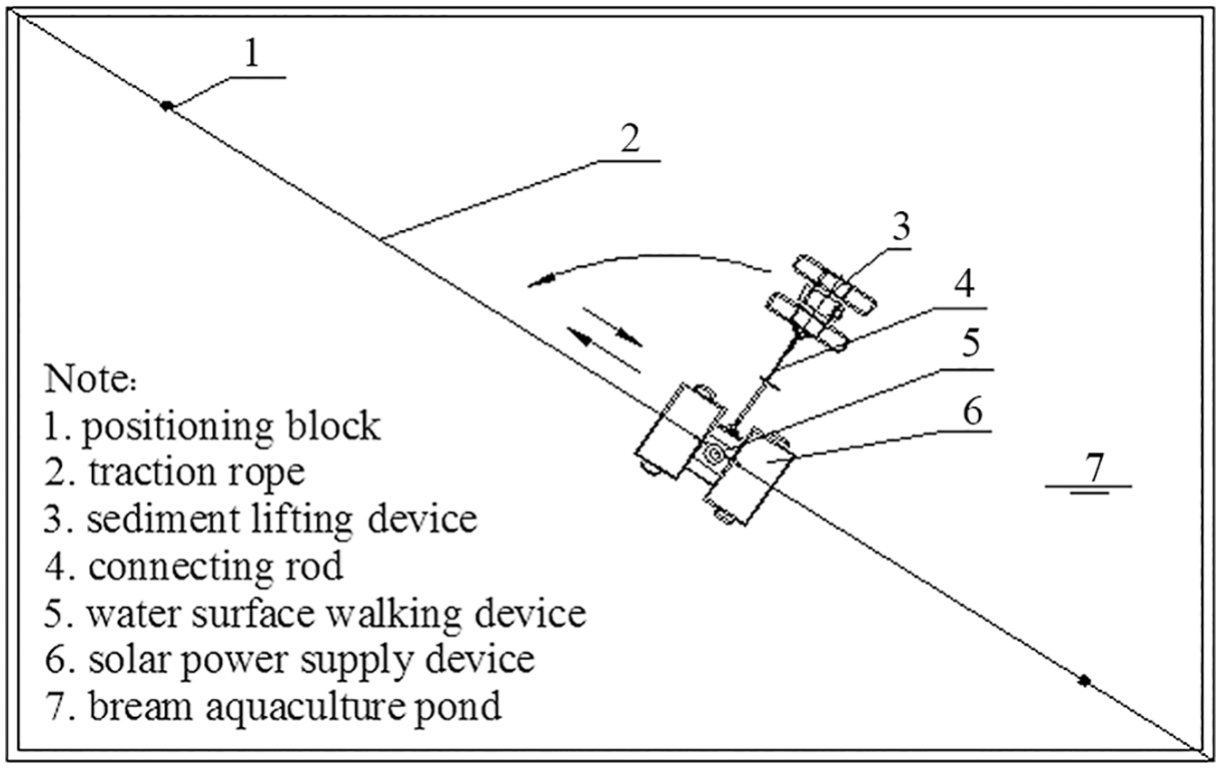
Processing craft of the SMWM.

A traditional Chinese bream culture pond is 0.5 hm^2^ to 2 hm^2^ in area and has a rectangular shape and a water depth of 1.2 m to 2.0 m [15]. The SMWM consists of a solar power supply device, a sediment lifting device, a mechanism for walking on the water’s surface and a control system. The solar power supply is the general power source of the SMWM. The sediment lifting device absorbs and releases the flocculent sediment and drives the sediment lifting device walking around the solar power supply device operation. The mechanism for walking on the water’s surface causes the machine to move back and forth across the water in a specific direction. The control system is responsible for the illumination start-up and remote control. All of the units are borne by the floating body, the buoyancy of which is greater than 120% of the weight of the units it bears (Fig 1).

### 2.2 The SMWM design and manufacturing process

#### 2.2.1 Sediment lifting device

Design theories: Based on the pond’s structure and convenience of operation, the sediment lifting device is designed with a telescopic regulating function, which has a telescopic range from 0.5 to 2.0 m. To ensure that the water quality is controlled effectively, the flux through the sediment lifting device is designed to be not less than 120 m^3^/h, and the head of the pump is no higher than 0.3 m The power of the sediment lifting device’s was shown the following [16]:

$$p_{e}=\frac{\rho gQH}{\eta }$$ (1)

where *P*_*e*_ represents the motor power of the Sediment lifting device, *ρ* is the density of water (1 x 10^3^ kg/m^3^), *g* is the gravitational acceleration (9.8 m/s^2^), Q is the total lifting capacity (120 m^3^/h), *H* is the height of the head of the sediment lifting device (0.3 m), and *η* is the dimensionless coefficient representing the total power (0.6).

As determined from Eq 1, the required motor power is 167 W. The actual motor power should generally be greater than the theoretical value; therefore, a DC motor that is rated for a power of 200 W at 240 rpm was built.

**Fig 2:**
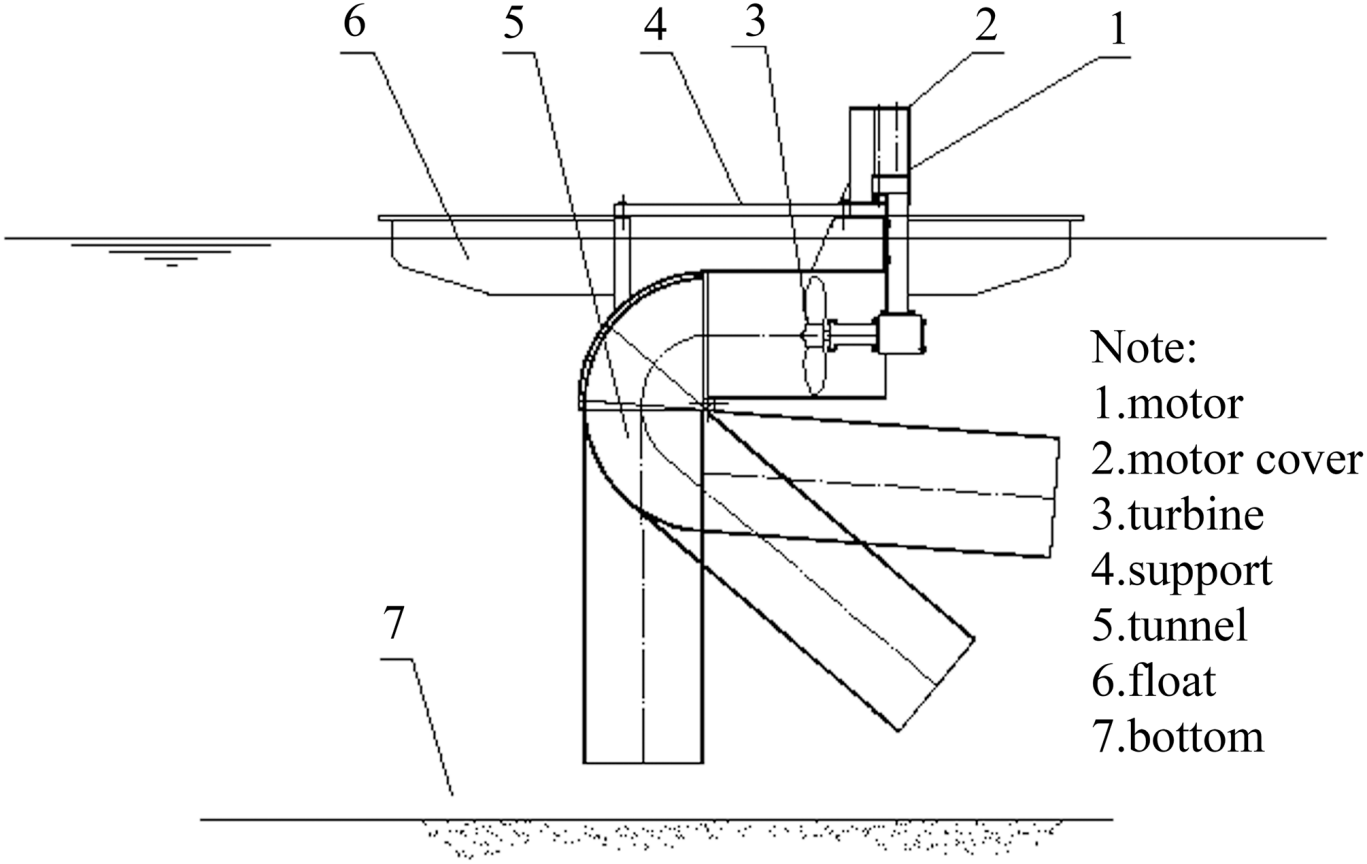
Structure of the sediment lifting.

Making method: The sediment lifting device was designed activity joints as a long circular pipe made from stainless steel. The structure has three sections and a telescopic length of 0.5 to 2 m (Fig 2). For stable operation, the equipment’s speed is controlled by the solar power supply through the stabilizer.

#### 2.2.2 Solar power supply device

Design theories: The solar power supply device primarily provides power for the sediment lifting device, walking mechanism, stabilizer, and light controller. Its total rated power is approximately 250 W. Using the amount of solar energy available in eastern China and the load parameters, the solar panel power of the SMWM was calculated, and the following results were obtained [16]:

$$P=\frac{p_{L}t}{H\eta_{2}}$$ (2)

where *p*_*L*_ is the total load power in W, *t* is the load work time (6.0 h), *H* is the average length of the daily radiation in h, and ƞ_2_ is the dimensionless charge loss (0.7). The input of these parameters into Eq 2 yielded a result of 400 W.

**Fig 3:**
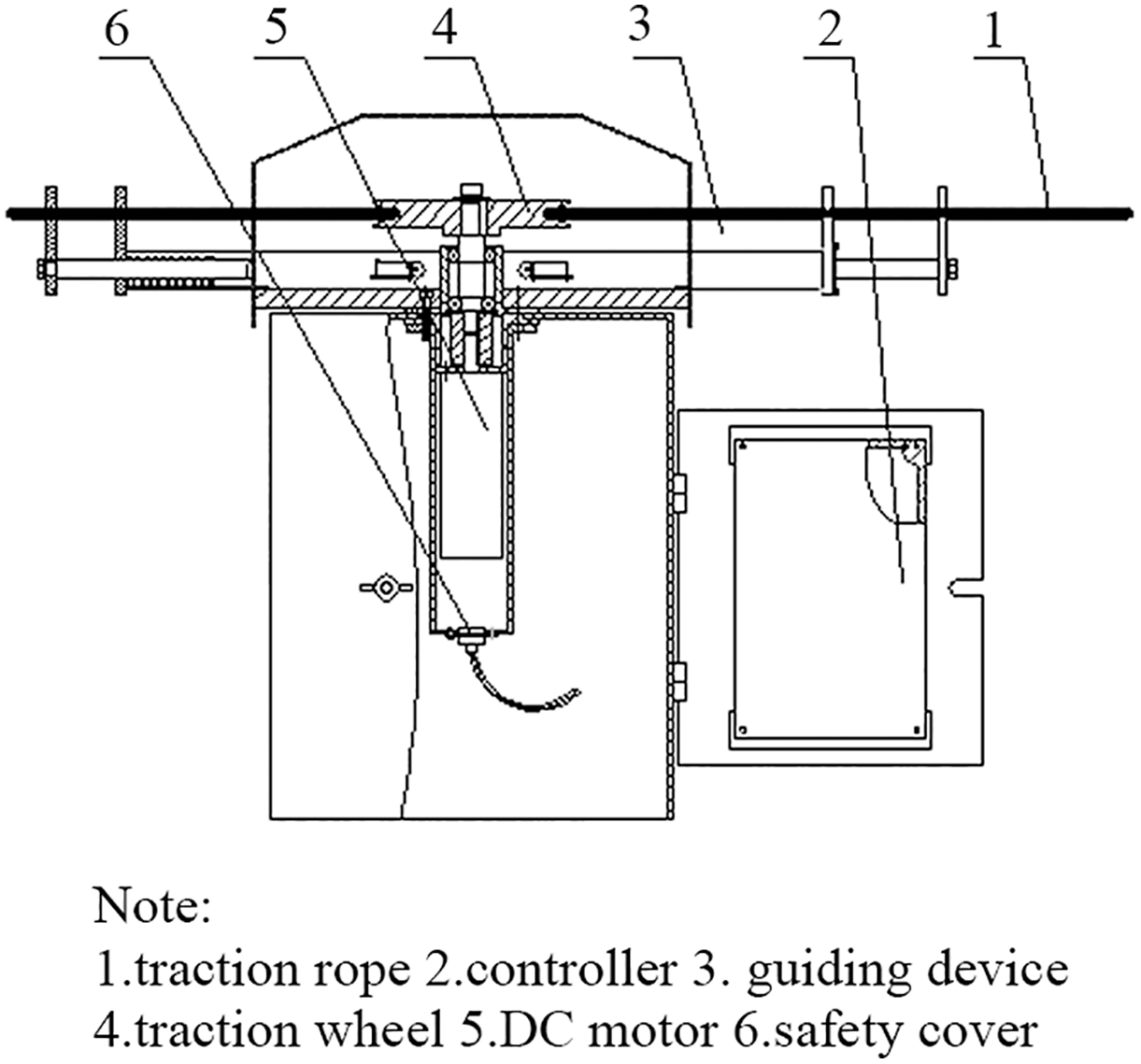
Structure of the water walking device of the SMWM.

Making method: The solar power system consists of two solar photovoltaic panels, a bracket, and a power output device installed on the floating body (Fig 3). Two photovoltaic panels (1.6 m x 0.8 m) offer power in parallel and are installed in two boat-shaped floating bodies with a bracket for balance. The output voltage of each photovoltaic panel is 24 V, and the highest working current is 9.1 A.

#### 2.2.3 Walking mechanism

Design theories: If the sunlight is sufficiently intense, friction will occur between the traction rope and the belt that rotates the wheel of the motor. This friction drives the SMWM’s run along the traction rope. There are two adjustable positioning blocks on the traction rope, and when the SMWM reaches a positioning block, a movable guiding rod presses a micro switch, which triggers the positioning block control module to makes the motor rotate in reverse. The SMWM moves back and forth along the traction rope.

**Fig 4:**
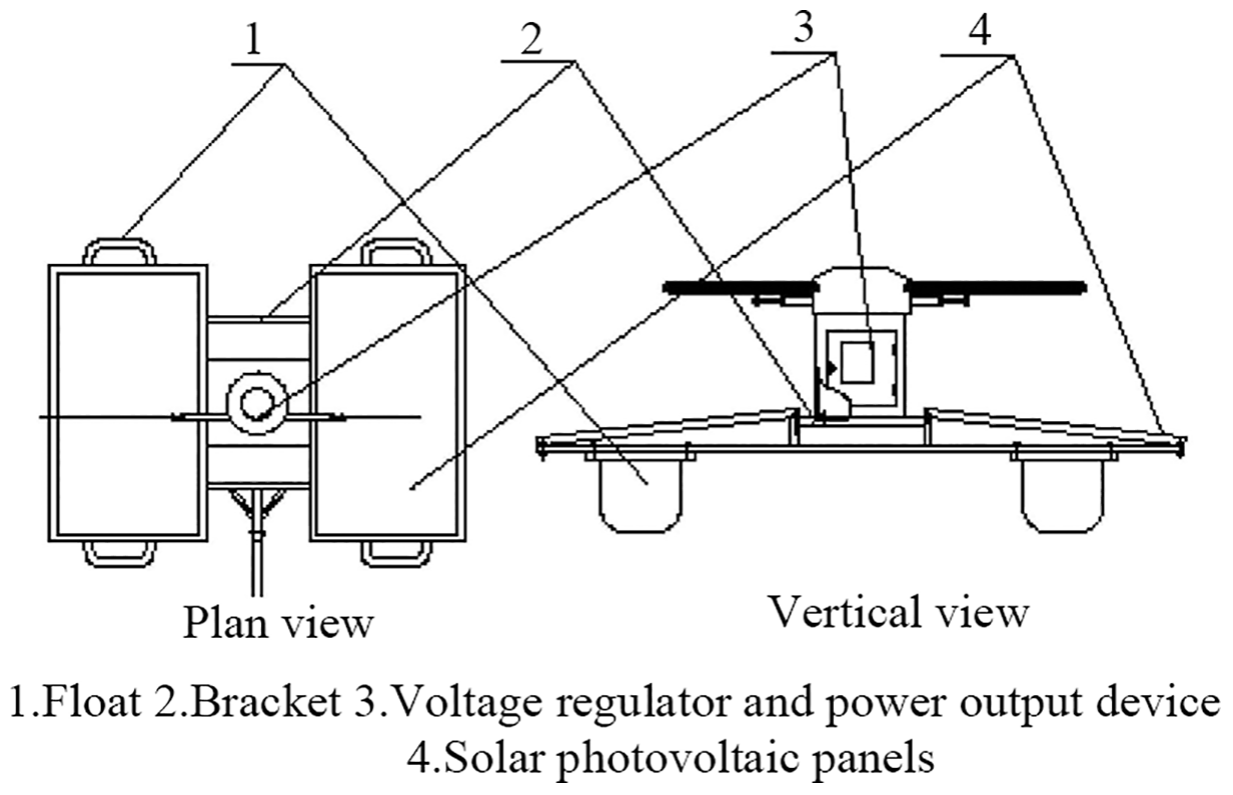
Structure of the walking mechanism of the SMWM.

Making method: The walking mechanism consists of a traction rope, a traction wheel, a DC motor, a guiding device, a controller module and a safety cover (Fig 4). The speed of the SMWM designed ranges from 0.02 to 0.03 m/s, the DC motor operates at 5 r/min, the traction wheel diameter is 150 mm, and the diameter of the traction rope is 2 mm.

#### 2.2.4 Control system

Design theories: To make the SMWM operate automatically under certain light conditions and to make flexible control unnecessary, the SMWM was designed with automatic light control and remote control functions. The illumination at which the maximum respiratory capacity reaches a rough balance with the oxygen production in bream culturing ponds in eastern China was estimated to equal 15,000 Lx [17], and the threshold for the SMWM’s light control system was set to 13,000 Lx. The SMWM should have light-dependent and remote control functions based on the requirements of the aquaculture pond. in sufficient light, the SMWM runs itself according to its programming. Remote control, which is realized through the light control and start/stop circuits, is available to facilitate aquaculture management.

**Fig 5:**
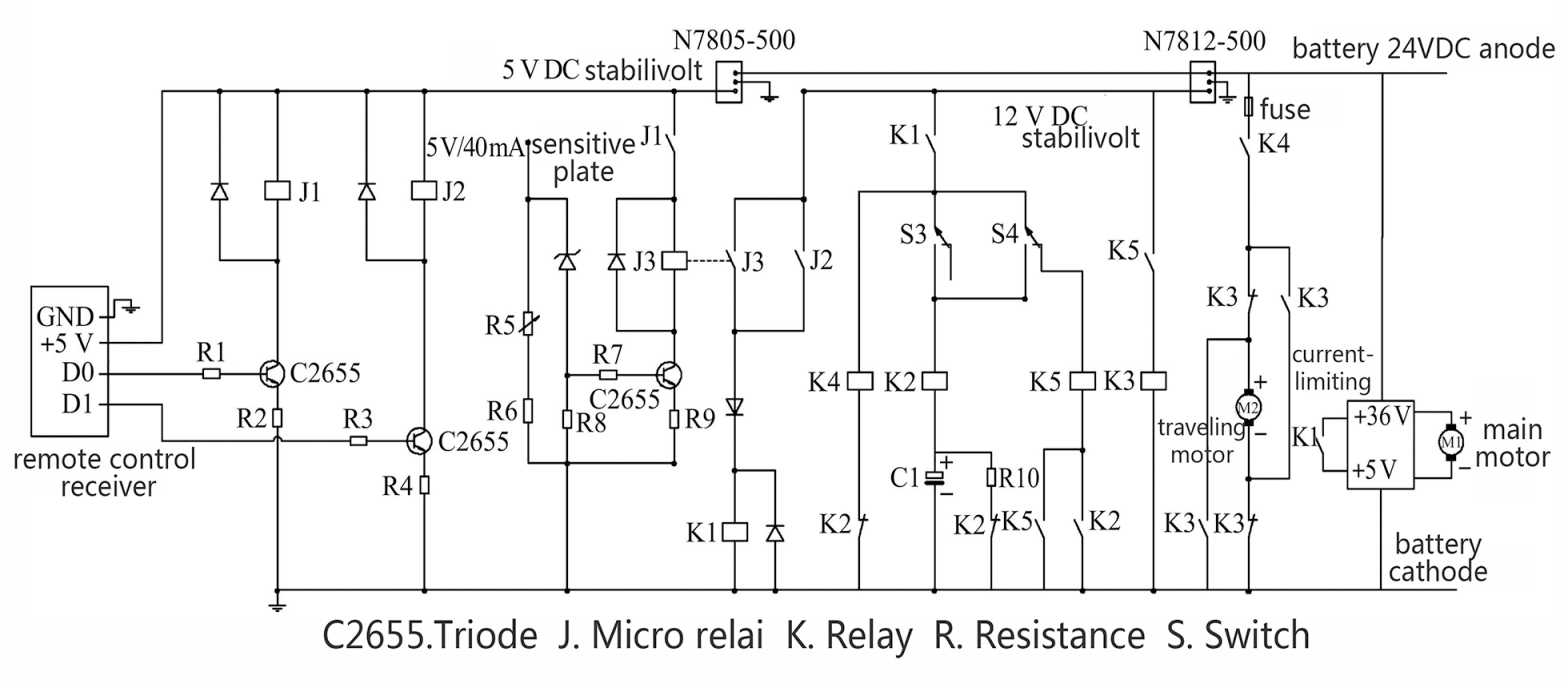
Sketch map of the working control system of the SMWM.

Making method: The light control circuit is primarily composed of diodes, triodes (C2655), micro relays (J), and resistor (R). The micro-relays are normally open as the output contacts of the light control circuit. The light control circuit’s output contact is closed when the illumination intensity exceeds a threshold. The light control circuit’s output contact and the remote control switch S2 are connected in parallel, and both are connected in series with the remote control switch S1, creating a start/stop control circuit that controls the device. The remote control switch S1 is the main switch, and S2 is as a standby switch for the light control circuit’s output contact. The SMWM begins to run when the light control circuit’s output contact or switch S2 closes as well as when the main switch S1 closes and when the start/stop control circuit connects. Relay K1 is charged after the start/stop control circuit begins to conduct electricity, its normally open contacts close, the circuit of the main motor conducts electricity, and the main motor begins to work. The return motor circuit then conducts electricity, the return motor begins to work, and the device runs over the water along the traction rope. The device moves to the limit at one end of the rope and touches the limit switch S3, the normally open contacts of which close, instantly electrifying relay K2. Its normally open contacts close, its normally closed contacts disconnect, the return motor loop disconnects, the main motor stops rotating, and the capacitor begins to charge. When relay K3 begins to charge, its normally open contacts close, and its normally closed contacts disconnect. K3 is locked and remains electrified. After approximately 1 s, the capacitor charging is complete, the relay K2 circuit disconnects, and its normally closed contacts close. At this time, because K3 keeps the device turned on, the return motor’s direction of rotation is changed, and the device runs along the traction rope in the opposite direction (Fig 5).

### 2.3 The SMWM tested methods

The mechanical properties of the SMWM were tested in a water tank at the Chinese Fishery Machinery Equipment Quality Supervision and Inspection Centre.

The SMWM’s work light intensity, amount of water elevated, no-load noise, speed across water, sediment lifting speed, wireless remote control distance, and other functions were tested by the China National Fisheries Machinery and Instrument Quality Supervision and Inspection Centre.

The intensity of illumination was measured with a TES-1339R light meter. The noise was measured in accordance with standard GB/T3768-1996. The running speed of the SMWM were calculation with unit time running distance. The wireless remote control distance were measured on site distance using tape measure. The light intensity was determined using a Taiwan ES1332A (TES) light meter, and the current was measured using a Yulid (UNI-T) UT61A universal meter.

Measurement of the turbine speed: A collision with the cylinder wall occurred during each rotation of the turbine results in the production of sound. The time interval between collisions was measured with a stopwatch, allowing the speed to be calculated.

To calculate the amount of water, because the tunnel’s cross-section was circular and the tunnel was homogeneous in the circular section, three measurement points were selected, and the three-point velocity was measured using an YSI Doppler velocity meter. This velocity was taken as representative of the average velocity in the annular section of the outlet. The flow was, where *S*_i_ represents the ring area.

### 2.4 The experiment ponds and fish culture

At the Songjiang Pond Ecological Engineering Research Center in Shanghai, six bream aquaculture ponds that were identical in both structure and aquaculture method were chosen for trial operation. The area of each pond was 100 m (length) × 50 m (width) with an average water depth of 1.8 m. The species farmed in the ponds primarily include Wuchang bream (*Parabramis pekinensis*) (80%) and silver and bighead carp (*Hypophthalmichthys molitrix*) (20%). Before this study’s test was conducted, the aquaculture density of each pond was measured to be 0.4 kg/m^3^. The SMWMs were installed in three testing ponds, while the other three pond was used as the control pond. The test started on June 1, 2013 and ended on November 15, spanning 165 days in total.

### 2.5 Water sampling and measurements

From 1 June 2013 to 15 Nov. 2013, three sampling points along the SMWM’s track in the three used SMWM ponds were selected; one sample site was located at the centre of the pond, and the distance between each of the other two sample sites and the shore of the pond was approximately 4 m. The sampling methods and sites of control ponds are the same as that of experiment ponds. At each sample site, samples of the surface water (0.5 m depth) and hypolimnion water (1.5 m depth) of the ponds were collected once a day using a ZPY-1 water sampler. The ammonia (NH_4_^+^–N), Nitrite (NO_2_^−^–N), total nitrogen (TN), total phosphorus (TP), available phosphorus (AP), Chemical Oxygen Demand (COD), and total suspended substance (TSS) concentrations were measured. From 1 June 2013 to 15 June 2013, samples were taken every day. From June 16, 2013 to November 15, 2013, samples were taken every a month, and all of the indicators were evaluated according to the protocols described in Water and Wastewater Monitoring Analysis Method [18]. In this document states that ammonia nitrogen (NH_4_^+^–N) must be measured using Nessler's reagent spectrophotometry; nitrite nitrogen (NO_2_^−^–N) must be measured using N-(1 naphtha)-ethylenediamine spectrophotometry; TN must be measured using the potassium-per-sulphate oxidation of ultraviolet spectrophotometry; TP must be measured using antimony-molybdenum spectrophotometry; COD_Mn_ must be measured using the acid method; and the available phosphorous (AP) must be measured using the sodium bicarbonate-molybdenum antimony colorimetric method.

### 2.6 Sediment sampling and measurements

The thickness of the sediment was tested using a PSC-700 sediment sampler. During the test period, sediment samples were taken every 30 d. The TN and TP of the sediment were determined according to China National Standard GB 9837–1988. The labile phosphorus of the pore water in the sediments was determined using molybdenum blue [19].

### 2.7 Data processing

Statistics were performed on the water index and production testing data with different software packages, including Excel 2010 and SPSS 19.0. Multiple comparison tests were conducted using Paired—Samples T test (*P*<0.05) and One-Way ANOVA (*P*<0.05) method.

## Results

### 3.1 Performance of the SMWM

#### 3.1.1 Operation station

On May 25, 2013, the mechanical properties of the SMWM were tested in a water tank at the Chinese Fishery Machinery Equipment Quality Supervision and Inspection Centre. The results showed that when the SMWM began to run, dark-brown water gushed from the sediment lifting device when the light intensity exceeded 13,000 Lx. The connecting rod was very strong, and the sediment lifting device stagnated when the speed of the SMWM was greater than 0.05 m/s; adjusting the connecting rod showed that this was due to too much stress deformation. After observation, the best speed and current for the SMWM were determined to be 0.02–0.03 m/s and 3.5–6 A, respectively. To maintain a stable speed, a 24 V/1 A Monistat was installed, the speed of the return motor was set to 5/min, and the diameter of the traction wheel was replacement of 150 mm.

**Fig 6:**
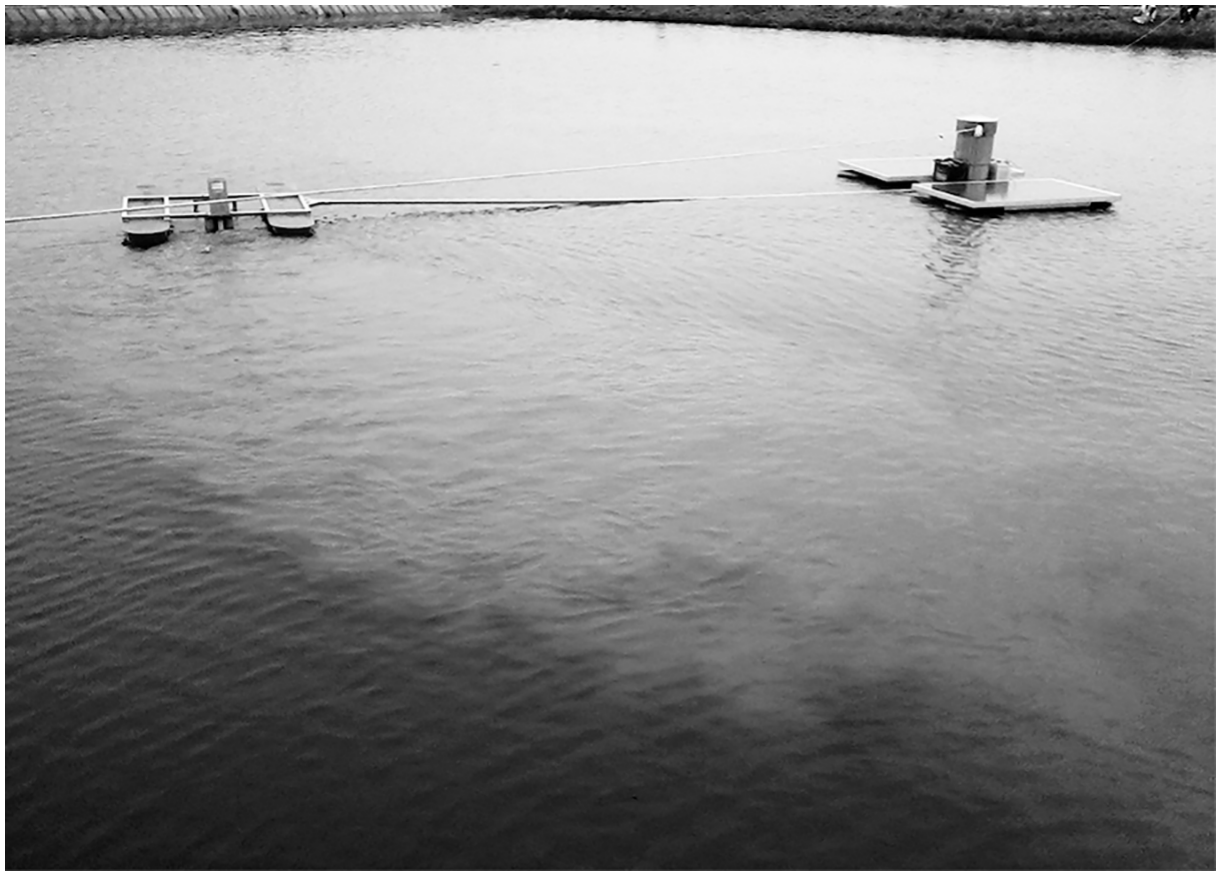
Performance of the SMWM.

The working range of the SMWM is closely related to the length of the connecting rod. The effective working width is approximately 12 m, and the work efficiency of SMWM can reach 600 m^2^/h. The observations revealed that if the link rod deforms easily if it is too long and exhibits low efficiency if it is too short. Therefore, the appropriate length of the link rod is between 4 and 8 m. Changing the position of the SMWM traction rope in different diagonal of the pond, could allows the working range to cover more than 80% of the pond (Fig 6).

#### 3.1.2 Effect of light intensity on the SMWM

On a sunny day (May 25, 2013), during the testing process, a simulated direct solar photovoltaic current was used to measure the influence of the current on the SMWM. The sediment lifting device was stretched to its maximum length. The results of the effect of light intensity on the SMWM in Table 1.

**Table 1:** Working status of the SMWM in different illumination.

luminance	Voltage	Current	Rotary speed	V_1_	V_2_	V_3_	Flow
Lx	V	A	R/M	m/s	m/s	m/s	m^3^/h
52500	20	8.5	200	0.723	0.688	0.388	207.95
42500	18	6.8	142	0.477	0.636	0.382	176.4
32500	16	5.1	125	0.385	0.551	0.32	145.5
30000	14.5	4.5	107	0.38	0.488	0.318	137.18
22500	12	3.8	100	0.331	0.421	0.284	119.81
12500	9.8	3.2	90	0.222	0.352	0.218	90.57

**Fig 7:**
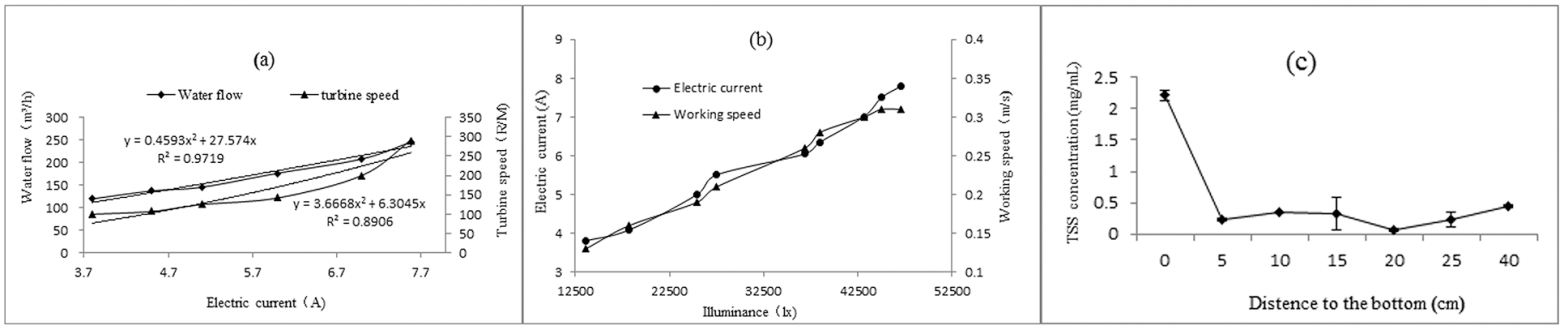
Effect of light intensity and entrance distance on the SMWM. (a) The relationship of illumination with turbine speed and water flow. (b) The relationship of illumination with current output and working speed. (c) The effect of floc sludge absorption relationship of the distance the sediment lifting device to the sediment’s surface.

The results showed that when the turbine started to run, the water flow rate of the sediment lifting device was approximately 90 m^3^/h when the light intensity was below 10,000 Lx. The translational speed of the SMWM continued to increase with an increase in the light intensity. The output current of the solar panel also increased as the illumination gradually increased (Fig 7a). The current output reached 8.5 A, the working speed of the sediment lifting device reached 0.35 m/s, and the water flow rate reached 280 m^3^/h when the illumination reached up to 52,500 Lx (Fig 7b).

Excessive absorption and release sediment can cause turbidity in aquaculture water, which may affect the safety of the cultured fish. The appropriate length of the sediment lifting device was 400 mm, which is conducive to water intake and flow control.

#### 3.1.3 Effect of sediment absorption

On May 25, 2013, the effect of floc sludge absorption by the SMWM was studied by adjusting the distance (5 cm, 15 cm, 20 cm, and 30 cm) between the entrance of the sediment lifting device and the sediment’s surface based on the concentration of the total suspended solids (TSS) in the water at the outlet. The results showed that the TSS concentration increased as the distance decreased. The TSS concentration reached 2300 mg/L at a distance of 5 cm. However, due to the uneven bottom of the pond, gravel, vivipara, and clams, among other particulates, were easily sucked into the cavity of the sediment lifting device, which could stop its turbine from rotating. It was found that the optimal distance between the entrance of the sediment lifting device and the sediment surface was 10–15 cm (Fig 7c).

#### 3.1.4 Control functions and operating range

Testing revealed that the SMWM could flexibly operate under automatic control in most cases and that the remote control distance of the SMWM was 50 m.

### 3. 2 Influence of the aquaculture pond water quality

From June 1 to 15, the SMWM in the ponds operated for 54 hours (i.e., 15 days) in total. Based on the water-quality inspection results, the concentrations of NH_4_^+^–N, NO_2_^−^–N and TN in the water were 0.202±0.001 mg/L, 0.012±0.003 mg/L and 3.133±0.379 mg/L, respectively, at the beginning of the test and increased rapidly with the operation of the SMWM. After a cumulative SMWM operation of 12 hours (i.e., 3 days), the concentrations of NH_4_^+^–N, NO_2_^−^–N and TN in the water increased to 0.649±0.013 mg/L, 0.128±0.008 mg/L and 6.1±3.346 mg/L, respectively, while those in the water body of the control pond were 0.282±0.002 mg/L, 0.018±0.003 mg/L and 3.50±0.563 mg/L, respectively, during the same period. Subsequently, the concentrations of NH_4_^+^–N, NO_2_^−^–N and TN in the water gradually reduced and became steady, showing that the operation of the SMWM could quickly increase the nitrogen content in the water. Consistent with the change of ammonia nitrogen in the water body, the concentration of TP and AP in the water of the ponds began to increase gradually from initial values of 1.51±0.141 mg/L and 1.23±0.122 mg/L to final values of 2.21±0.773 mg/L and 1.63±0.587 mg/L, respectively, during SMWM. After 15 hours (i.e., 4 days) of operation, the concentration of TP and AP began to decrease; these values were found to be different from those in the water of the control pond by approximately 10.28% and -139.53%, respectively. During the first 15 days of SMWM operation, the concentration of COD_Mn_ and TSS in the water of the testing ponds increased gradually from initial values of 41.667±3.215mg/L and 47.312±3.931 mg/L to 53.376±4.235 mg/L and 52.516±4.865 mg/L, respectively, and became steady after 12 hours. Compared to those in the water of the control pond, the concentration of COD_Mn_ in the water of the testing ponds increased by 28.1%. These results indicate that the operation of the SMWM could increase the concentration of pollutants in pond water.

**Fig 8:**
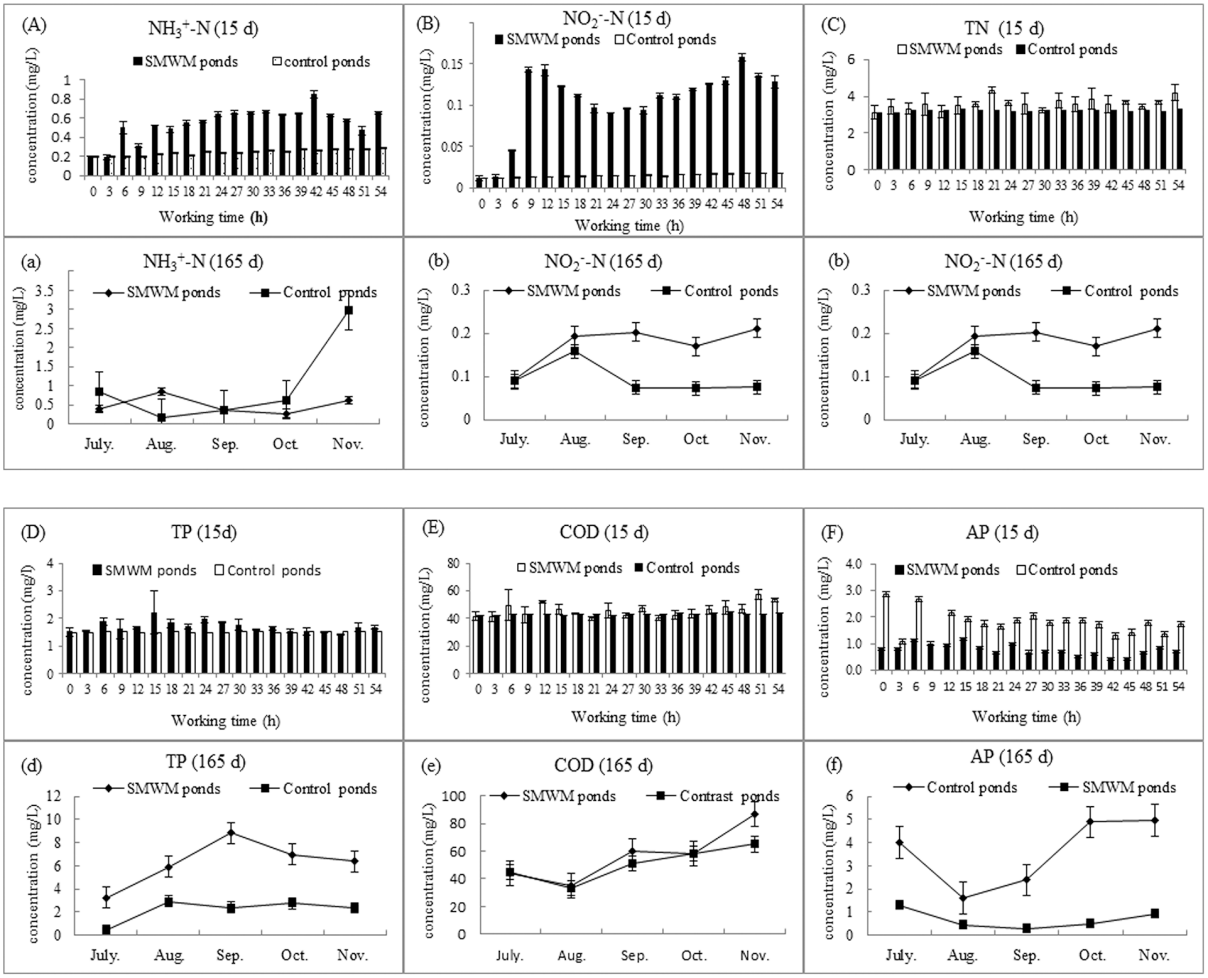
The influence of SMWM on bream culture pond water quality. (A),(B),(C),(D),(E) and (F) were the concentrations of NH_4_^+^–N, NO_2_^−^–N, TN, TP, COD_Mn_ and AP in used SMWM ponds and control ponds water at the beginning 15 days (From June 1 to June 15, 2013 the SMWM operation 54 hours). (a),(b),(c),(d),(e) and (f) were the concentrations of NH_4_^+^–N, NO_2_^−^–N, TN, TP, COD_Mn_ and AP in used SMWM ponds and control ponds water in 165 days (From June 1, 2013 to November 15, 2013).

From June 1, 2013 to November 15, 2013, The NH_4_^+^–N, NO_2_^−^–N, TN, TP, AP, COD_Mn_, and TSS concentrations of the aquaculture water were analysed throughout the experiment. The results show that the NH_4_^+^–N, NO_2_^−^–N, TN,TP, AP, COD_Mn_, and TSS concentrations in the ponds with SMWMs increased rapidly at the beginning, and during the experiment, the mean concentrations of NH_4_^+^–N, NO_2_^−^–N,TN, TP, AP and COD_Mn_ in the ponds with SMWMs were 0.821±0.142 mg/L, 0.203±0.07 mg/L, 4.08±1.033 mg/L, 6.26±2.018 mg/L, 0.762±0.413 mg/L and 56.8±19.766 mg/L, respectively, whereas the corresponding concentrations in the control ponds were 2.975±1.07 mg/L, 0.158±0.06 mg/L,4.28±1.11 mg/L, 2.164±1.010 mg/L, 3.572±1.510 mg/L and 50.4±12.280 mg/L, respectively (Fig 8). These results showed that the use of SMWMs in bream culture ponds can decrease the NH_3_^+^–N and AP concentrations (Paired—Samples T test, *P*<0.05) and improve the TP, TN concentration (Paired—Samples T test, *P*<0.05) of the water but did not change the TN and COD concentrations.

### 3. 3 Influence of sediment

#### 3.3.1 Influence of the sediment’s structure

Before the experiment, the sediment thickness along the ponds’ diagonal direction was measured has a thickness of 35.2±7.5 cm, was black in colour, and it gives off a very unpleasant smell. At the end of the experiment, using the same technique, the sediment in the ponds with SMWMs has a thickness of 22.7±5.6 cm thick, corresponding to a decrease of 12.4±4.2 cm, and the sediment in the control ponds had a thickness of 40.3±9.7 cm thick. In the winter, all of the bream culture ponds were drained and cleared, and we found that the bottoms of the ponds in which SMWMs had been used were lighter in colour and taste, whereas the bottoms of the control ponds were black and smelled bad.

#### 3.3.2 Influence of the TN and AP concentrations of the sediment

The TN and AP concentrations of the sediment throughout the experiment were analysed. The results shown that the TN and AP concentrations of the bream pond sediment were 1.150±0.061 g/kg and 18.012±3.509 mg/kg, respectively, before an SMWM was used. With the SMWM operating, the concentrations of TN and AP in the sediment reduced gradually. After 54 hours (i.e., 10 days), the concentrations of TN and AP reduced to 1.023±0.001 g/kg and 14.567±4.310 mg/kg, respectively, compared to the original sediment sample, indicating that SMWM operation could accelerate the release of TN and AP (Paired—Samples T test, *P*<0.05) in the bottom sediment.

**Fig 9:**
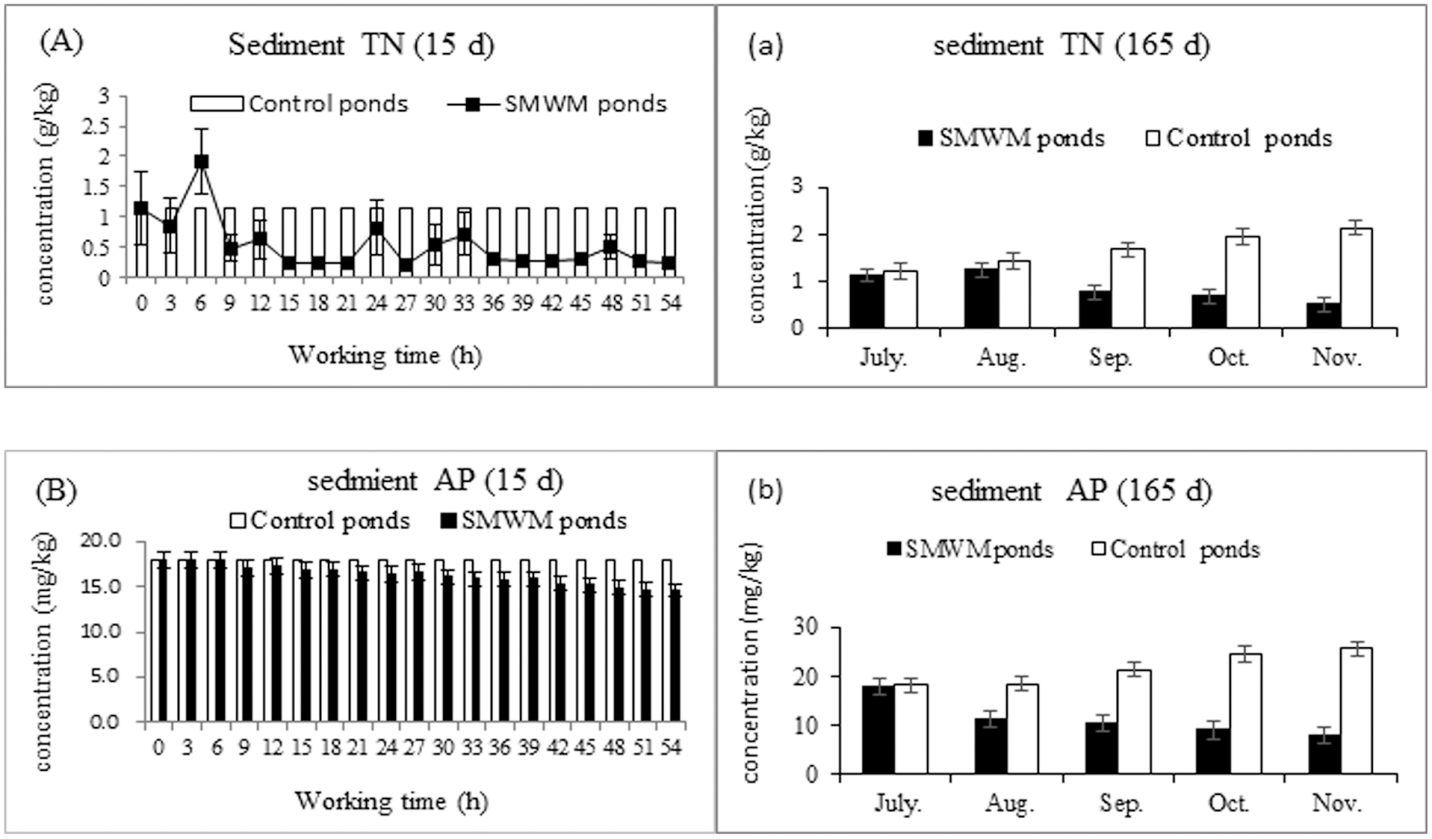
Influence of SMWM on bream culture pond sediment. (A) and (B) were the concentrations of TN and AP in used SMWM ponds and control ponds seiment at the beginning 15 days (From June 1 to 15, 2013 the SMWM operation 54 hours). (a) and (b) were the concentrations of TN and AP in used SMWM ponds and control ponds sediment in 165 days (From June 1, 2013 to November 15, 2013).

At the end of the experiment, the TN and AP concentrations in the ponds in which SMWMs were 0.520±0.121 g/kg and 8.023±1.221 mg/kg, respectively, whereas in the control ponds, the concentrations were 2.150±0.094 g/kg and 25.634±2.509 mg/kg, respectively. These results show that the use of SMWMs in bream ponds could reduce the nitrogen and phosphorus contents of the sediment and reduce the pollution due to sediment deposition (Fig 9).

### 3.3 Influence of production

On Nov. 15, 2013, we caught all of the fish in the experimental ponds and measured them (Table 2). As shown in Table 1, in the ponds in which SMWMs were used, the Wuchang bream and silver carp produced weighed 6975.3±123.7 kg and 383.5±12.5 kg, respectively, and the stocking density was 0.84±0.07 kg/m^3^. The Wuchang bream and silver carp from the control ponds weighed 5281.7±143.5 kg and 305.6±15.2 kg, respectively, and the fish breeding density was 0.67±0.05 kg/m^3^. The Wuchang bream and silver carp production in the ponds in which SMWMs were used increased by 32.1% and 24.7%, respectively, compared with that observed in the control ponds. In addition, the fish stocking density increased by 25.37%, and the fish feeding coefficient decreased by 24.24%. Statistics results shown that the production of bream and silver carp, end size, feed coefficient and stocking density in used SMWM ponds were significantly higher than control ponds (One-Way ANOVA, *P*<0.05).

**Table 2:** Production of the experimental ponds.

Ponds	Index	Silver carp	Wuchang bream
SMWM ponds	Output (kg)	383.5±12.5^A^	6975.3±123.7^D^
	Initial average weight (kg)	0.125±0.031	0.072±0.006
SMWM ponds	End average weight (kg)	1.513±0.726^B^	0.688±0.534^E^
	Feeding quantity (kg)		12200
	Feed coefficient		1.75^F^
	Output (kg)	305.6±15.2^a^	5281.7±143.5^d^
	Initial average weight (kg)	0.125±0.031	0.072±0.006
Control ponds	End average weight (kg)	1.240±0.684^b^	0.604±0.087^e^
	Feeding quantity (kg)		12200
	Feed coefficient		2.31^f^

**Note:** The same letter in table with capital and lowercase shown there were significant differences (*P*<0.05)

## Discussion and Conclusions

Due to the lack of technology and equipment for regulating water quality and sediment in pond cultures, sediment pollution has been influencing biosafety and productivity during cultivation in China [3]. Therefore, we developed the SMWM in accordance with the ecological conditions of pond culture in China. The SMWM is characterized by its novelty and its unique design.

Numerous studies have indicated that sunlight is directly related to the photosynthesis of algae in water [20, 21]. Yao et al. investigated ponds for culturing Wuchang bream in places such as Nanjing, Yangzhou, and Suzhou in China. Because the oxygen produced through photosynthesis in a 1-m column of water per day (PG) was between 3.00 and 28.92 g/(m^2^ d) and the oxygen consumed by respiration per day (Pw) was between 1.86 and 19.50 g/(m^2^ d) [17], the illumination at which the maximum respiratory capacity reaches a rough balance with the oxygen production in ponds for culturing bream in eastern China was estimated to equal 15,000 Lx. It has been reported that the total amount of solar radiation is between 3340 and 8400 MJ/m2•y with a median of 5852 MJ/m^2^•y in China, and the solar radiation of the eastern central region of China is between 5016 and 5852 MJ/m^2^•y, which is equivalent to the heat emitted by the combustion of 170 to 200 kg of standard coal. The average length of the daily radiation is 4.5 hours during the culture period, which lasts from June to September [22]. In our study, the starting illumination of the regulator was set to 13,000 Lx, which met the demands of both all of the electrical equipment needed to start a current and the organisms’ need for oxygen for oxidation and decomposition. The experimental results showed that the pond did not lack oxygen when the regulator was operated at above 13,000 Lx. In addition, the regulator’s sediment absorption rate varied with changes in the illumination because it was able to make the best of the illumination provided and improve the sediment oxidation and decomposition. Because the SMWM moves in two directions, it can operate in more than 80% of the pond’s surface. The SMWM solved the problem of the fixed pollution regulator. The SMWM supports both light-based and remote control, in contrast to other cultivation regulators. With light-based and remote control, the operator can run the SMWM with a more flexible manner to meet the demands of production management. In addition, by virtue of the unique cavity design, which includes activity joints in the device for absorbing and releasing flocculent sediment, it is feasible to absorb and release sediment in the pond at a depth of 0.5–2.0 m or in the water exchange layer. These features are the characteristics of this device.

In this study, in the ponds in which the SMWM was used, the concentrations of NH_3_^+^–N and AP in the water were significantly lower than those in the control ponds(Paired—Samples T test, *P*<0.05). The decrease in the concentration of NH_3_^+^–N may be related to the SMWM’s ability to improve the amount of dissolved oxygen, which ammonia ratio low under the oxygen-rich conditions [23]. During the test period, the concentration of the available phosphorus in ponds in which the SMWM was used remained low, but the concentration of TP was significantly higher than that observed in the control ponds (Paired—Samples T test, *P*<0.05), which may be due to the release of suspended solids or the adsorption of available phosphorus into the water from the interstitial water in the sediment.

Some studies have shown that the amount of phosphorus accumulated at the bottom of a culture pond can reach 2.3–3.0 tons per hectare [24]. Loosely dissolved phosphorus is easily discharged into interstitial water and then absorbed or released due to different physical and chemical effects [25]. During fertilization, the concentration of phosphorus in the water in a culture pond increases rapidly but soon decreases to its pre—treatment level because a significant amount of phosphorus is absorbed by algae and the sediment [26]. The change in the available phosphorus and the TP in the water in the ponds in which the SMWM was used in the study is consistent with the findings described above. Following the application of the regulator, the concentrations of COD and TSS in the ponds used for culturing Wuchang bream did not change significantly during the culture cycle, indicating that in using the SMWM did not cause water turbidity in the ponds. The structure of the sediment in the ponds and its nitrogen and phosphorous contents were significantly changed by the regulator, as expected.

The yield in the ponds for culturing bream in which the SMWM was used can improve substantially because the SMWM can change the structure and amount of nutrients, improve the primary productivity of the water, reduce the concentration of NH_3_^+^–N and other substances harmful to fish, and improve the environment on the bottom of the pond. Therefore, the SMWM not only improves the production of silver carp but also provides better conditions for growing bream fish.

To date, the SMWM has been used in Chinese provinces and regions such as Yunnan, Guangdong, Anhui, and Ningxia. Practice has shown that it is able to improve the pond sediment, regulate water quality, and increase culture efficiency, making it suitable for mixed aquaculture in ponds where freshwater fish are cultured for bulk sales.
